# Graphical Abstract: ChemistryOpen 5/2015

**DOI:** 10.1002/open.201580511

**Published:** 2015-10-08

**ChemistryOpen** is a multidisciplinary, gold-road, open-access, international forum for the publication of Reviews, Full Papers and Communications from all areas of chemistry and related fields. ChemistryOpen also publishes the Thesis Treasury containing summaries of Ph.D. theses and links to the full version via our homepage. Based in Europe, ChemistryOpen attracts authors and readers from around the world, as open-access publishing becomes more important in all areas of chemistry. ChemistryOpen is coowned by ChemPubSoc Europe and published by Wiley-VCH. Authors can submit their review articles, primary research articles and thesis summaries via our homepage by clicking “submit an article”. All contributions considered suitable for publication are subject to peer review, and if accepted, electronically processed and published online ensuring high quality and short publication times.

## COVER PICTURE

**The cover picture compares** the rearrangement of a small molecule to the process of turning a stuffed animal inside out. The recycled, inside-out stuffed animals are both artistic and philosophically provocative. They capture the essence of the rearrangement reaction because the compounds themselves turn inside out throughout the reaction, extending the diversity of products that can arise from simple starting materials. The epoxidation of some highly functionalized spiroketal compounds promoted rearrangements of their structures that turned them inside out. Some of the features of the products led the team to use X-ray crystallography and computerassisted structure elucidation, computation, and a new version of the 1,1-ADEQUATE NMR experiment to determine their structures. More information can be found in the Communication by Mark W. Peczuh et al. (DOI: 10.1002/open.201500122).


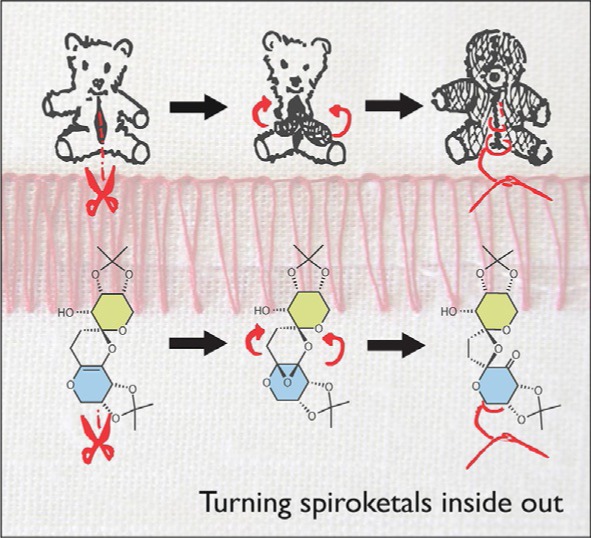


## COVER PROFILE

C. Lorenc, J. Saur., A. Moser, A. V. Buevich, A. J. Williams, R. T. Williamson, G. E. Martin, M. W. Peczuh*

**542 Turning Spiroketals Inside Out: A Rearrangement Triggered by an Enol Ether Epoxidation**

“There were several moving parts in this project: synthetic method development, computer-assisted structure elucidation, and new spectroscopic techniques supported by computational chemistry. Each team member brought something unique to the work.”

Learn more about the story behind the research featured on the front cover in this issue’s Cover Profile. Read the corresponding article on p. 577 ff.


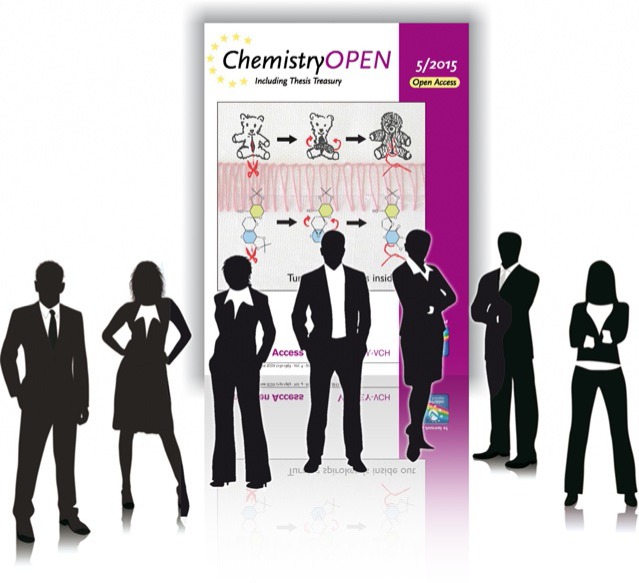


## NEWS

Spotlights on our sister journals

552 – 555

## REVIEWS

F. Coutrot*

**A Focus on Triazolium as a Multipurpose Molecular Station for pH-Sensitive Interlocked Crown-Ether- Based Molecular Machines**

**Magnificent molecular machines!** Triazolium has recently emerged as a multipurpose molecular station for crown ethers in a wide range of interlocked molecular architectures. Its easy access and specific ability to interact with crown ethers allowed for the design, synthesis, study, and utilization of several pH-sensitive molecular machines which are described in this review.


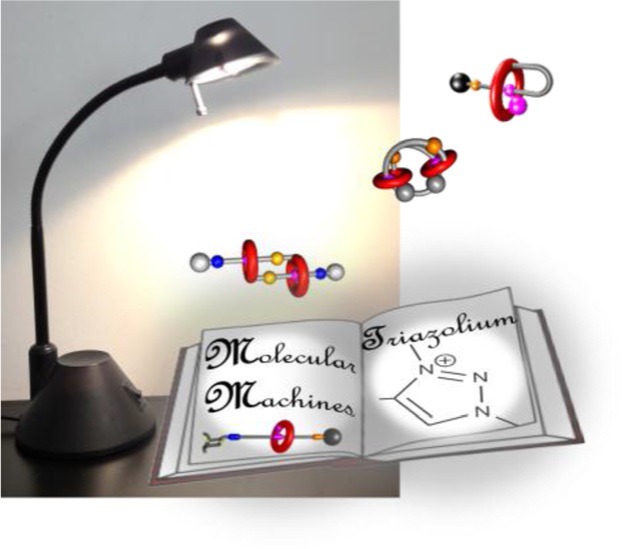


## COMMUNICATIONS

C. Lorenc, J. Saur., A. Moser, A. V. Buevich, A. J. Williams, R. T. Williamson, G. E. Martin, M. W. Peczuh*

577 – 580

**Turning Spiroketals Inside Out: A Rearrangement Triggered by an Enol Ether Epoxidation**

**Turn it inside out!** Epoxidations of polycyclic, enol-ether-containing spiroketals triggered rearrangements that completely remodeled their structures, essentially turning them “inside out”. Due to the high level of substitution on the carbon skeletons of the substrates and products, characterization resorted to X-ray crystallography and advanced computation and NMR techniques to solve their structures.


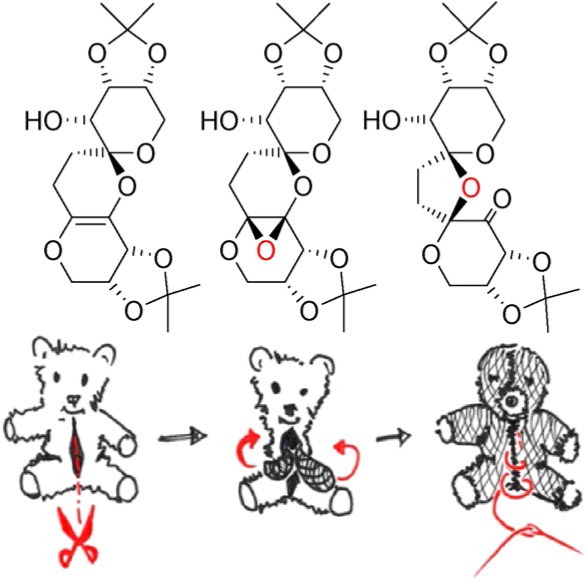


S. Okusu, K. Hirano, E. Tokunaga, N. Shibata*

581 – 585

**Organocatalyzed Trifluoromethylation of Ketones and Sulfonyl Fluorides by Fluoroform under a Superbase System**

**Fluoroform and superbase!** The organic-superbase-catalyzed trifluoromethylation of ketones and arylsulfonyl fluorides by HCF3 is described. Reactions were performed with a newly developed superbase organocatalyst system consisting of P_4_-tBu and N(SiMe_3_)_3_ to convert a series of ketones and arylsulfonyl fluorides into the corresponding a-trifluoromethyl carbinols and aryl triflones in THF or DMF. Protonated P_4_-tBu (H[P_4_-*t*Bu]^+^) is suggested to be crucial for the catalytic process.


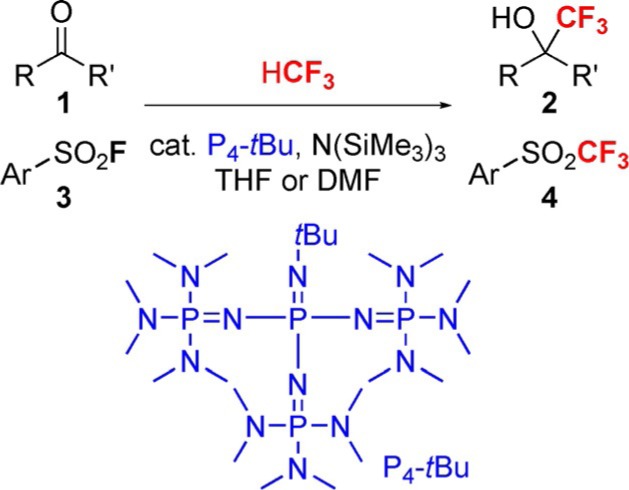


L. Jiang, G. W. Nelson, H. Kim, I. N. Sim, S. O. Han,* J. S. Foord*

586 – 589

**Cellulose-Derived Supercapacitors from the Carbonisation of Filter Paper**

**The power of paper!** Pure filter paper made from cellulose was successfully converted to a conductive carbon material by carbonising at different temperatures from 600 to 1700 °C. The material with the best specific capacitance with a high stability was obtained by carbonizing at 1500 °C. This carbonised filter paper, without addition of additives, is a promising alternative carbon material for supercapacitor applications.


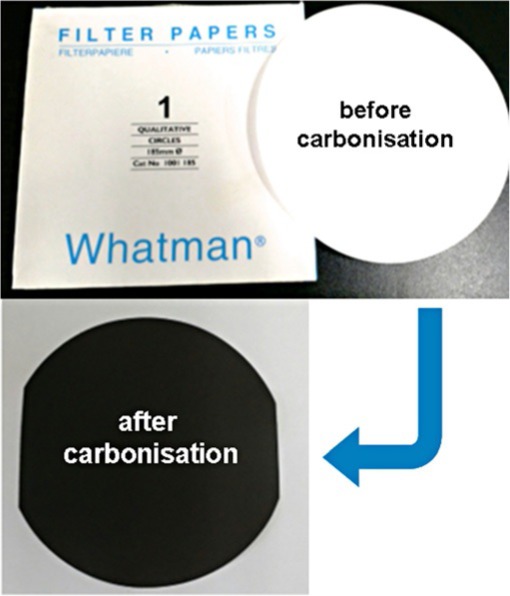


S. N. Anderson, J. M. Richards, H. J. Esquer, A. D. Benninghoff, A. M. Arif, L. M. Berreau*

590 – 594

**A Structurally-Tunable 3-Hydroxyflavone Motif for Visible Light-Induced Carbon Monoxide- Releasing Molecules (CORMs)**

**Inspired by nature!** A family of new 3-hydroxyflavone derivatives was prepared and found to exhibit quantitative carbon monoxide (CO) release upon illumination with visible light under various conditions. These compounds exhibit many features that are desirable in next-generation visible-light-induced CO-releasing molecules for potential biological applications.


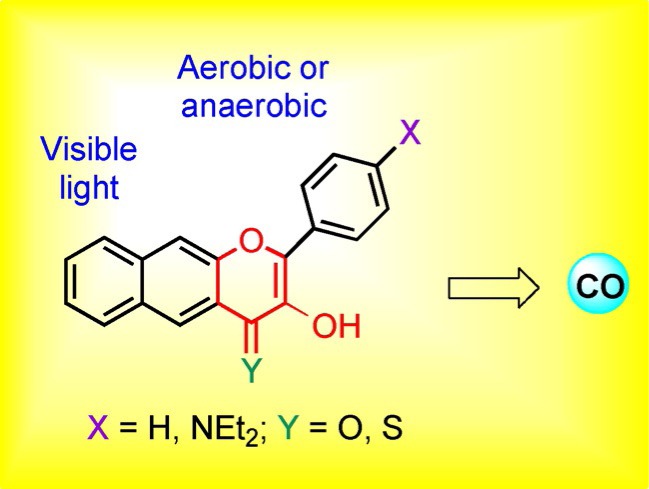


D. V. Navolotskaya, H. S. Toh, C. Batchelor--McAuley, R. G. Compton*

595 – 599

**Voltammetric Study of the Influence of Various Phosphate Anions on Silver Nanoparticle Oxidation**

**Influential phosphate!** The antibacterial properties of silver are strongly controlled by the silver/silver(I) redox couple. With the abundance of phosphate species in biological systems, this work reports the influence of phosphate anions on silver nanoparticle oxidation. The influence of phosphate anions on silver nanoparticle oxidation was determined to be: PO_4_^3–^ < HPO_4_^2–^ < H_2_PO_4_^–^.


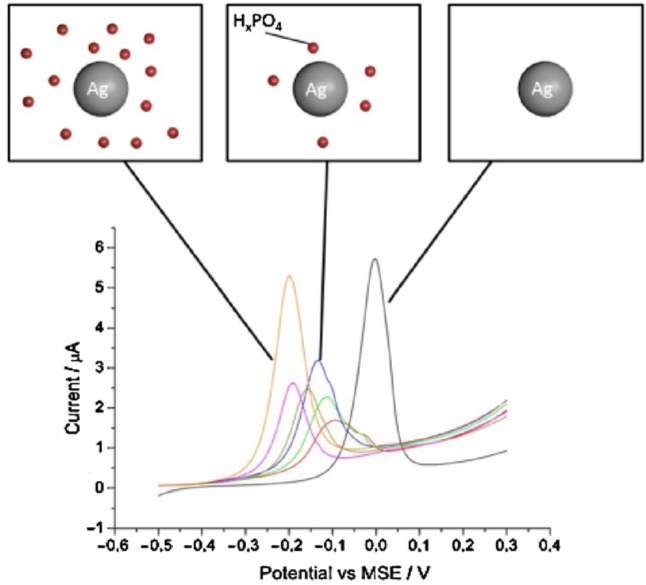


## FULL PAPERS

T. R. Bartlett, S. V. Sokolov, R. G. Compton*

600 – 605

**Electrochemical Nanoparticle Sizing Via Nano-Impacts: How Large a Nanoparticle Can be Measured?**

**Size it to the limit!** The ‘nano-impacts’ technique is shown to be an excellent and qualitative in situ method for nanoparticle characterisation, covering the range 4–100 nm. Two complementary studies on silver and silver bromide nanoparticles were used to assess the large radius limit of the nano-impact method for NP sizing.


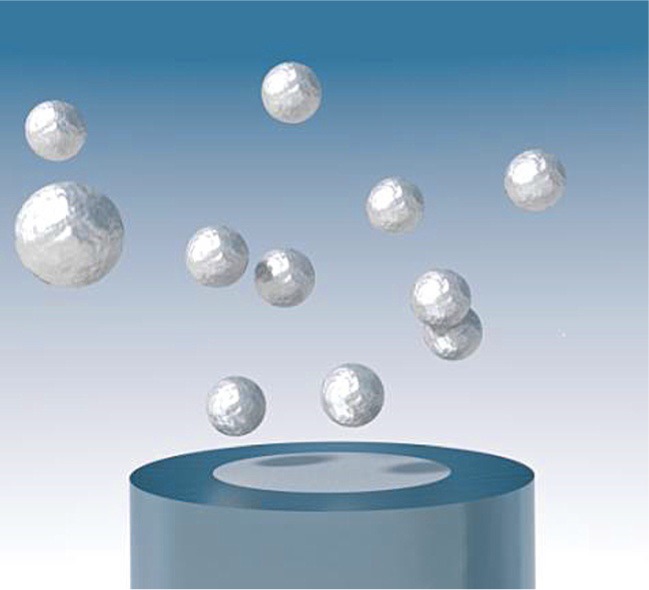


P. Gan, J. S. Foord, R. G. Compton*

606 – 612

**Surface Modification of Boron-Doped Diamond with Microcrystalline Copper Phthalocyanine: Oxygen Reduction Catalysis**

**Dropcasting on doped diamond:** Modification of boron-doped diamond with microcrystalline copper phthalocyanine by dropcast deposition was found to be sensitive to the surface termination. After modification of the hydrogen-terminated diamond, a significant electrocatalysis was observed for oxygen reduction, while this effect was not seen at the oxidised diamond.


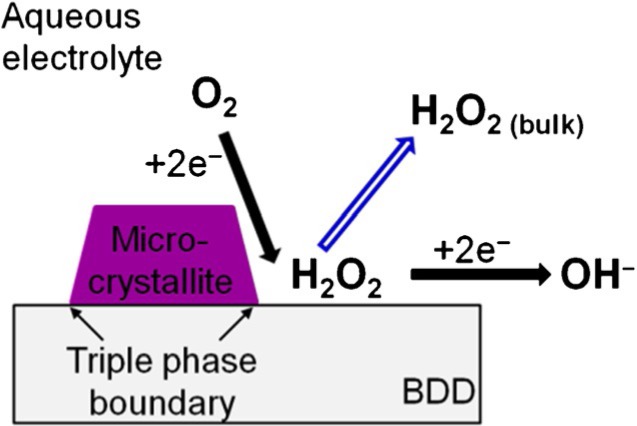


E. Agustina, J. Goak, S. Lee, Y. Seo, J.-Y. Park, N. Lee*

613 – 619

**Simple and Precise Quantification of Iron Catalyst Content in Carbon Nanotubes Using UV/Visible Spectroscopy**

**Purity assessment with phen:** A simple colorimetric system can be used to determine the iron catalyst content in carbon nanotubes (CNTs). Iron dissolution from CNTs was investigated with various acids, either alone or in mixtures; shown here are: a) HCl/HNO_3_ (3:1), b) H_2_SO_4_/HNO_3_ (3:1), c) HClO_4_/ HNO_3_ (3:1), and d) HClO_4_/fuming-HNO_3_ (3:1). The latter solution (d) completely dissolved the CNTs, rendering the sample suitable for analysis.


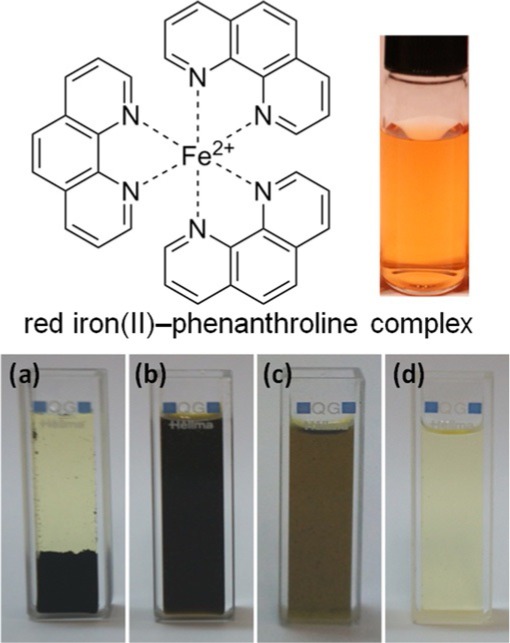


K. Refson, S. F. Parker*

620 –625

**Assignment of the Internal Vibrational Modes of C_70_ by Inelastic Neutron Scattering Spectroscopy and Periodic- DFT**

**Good vibrations!** Vibrational spectroscopy is a key tool in characterising fullerenes. For C70, we have obtained a new inelastic neutron scattering spectrum from a large sample recorded at the highest resolution available. We demonstrate that all previous assignments are incorrect in some respects and propose a new assignment based on periodic density functional theory that successfully reproduces the inelastic neutron scattering, infrared, and Raman spectra.


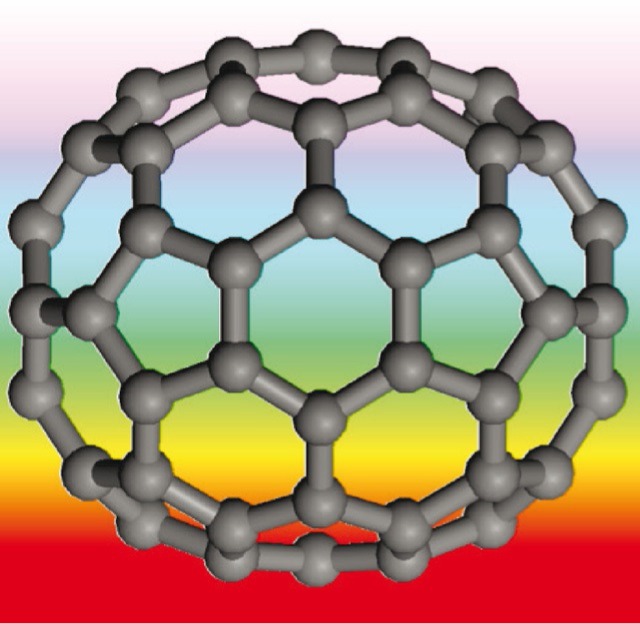


M. Kumar, L. K. Kumawat, V. K. Gupta, A. Sharma*

626 – 632

**2-(Alkylamino)-3-aryl-6,7- dihydrobenzofuran-4(5H)-ones: Improved Synthesis and their Photophysical Properties**

**Easy on the eyes:** A solvent-less, diversity enabling, high yielding, energy efficient one-step protocol has been devised to access 2-(alkylamino)-3-aryl-6,7- dihydrobenzofuran-4(5H)-one. Extensive photophysical studies to evaluate the absorption and fluorescence behavior of the synthesized derivatives revealed that two indole-containing furanones have potential as aluminum(III) chemosensors.


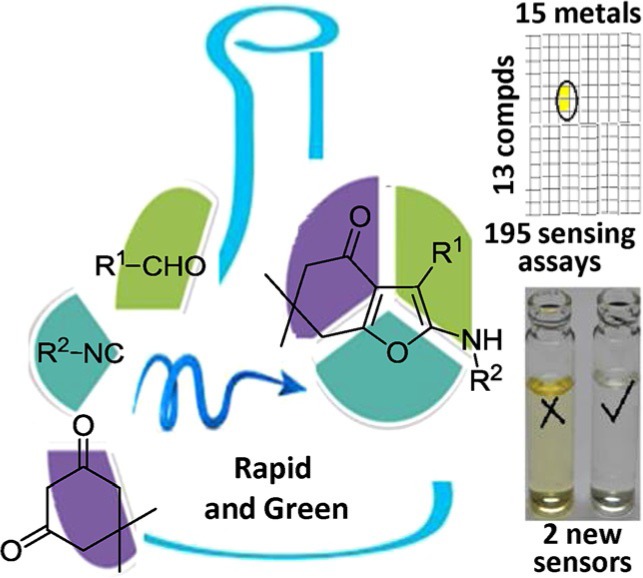


S. Zanella, M. Mingozzi, A. Dal Corso, R. Fanelli, D. Arosio, M. Cosentino, L. Schembri, F. Marino, M. De Zotti, F. Formaggio, L. Pignataro, L. Belvisi, U. Piarulli,* C. Gennari*

633 – 641

**Synthesis, Characterization, and Biological Evaluation of a Dual-Action Ligand Targeting avb3 Integrin and VEGF Receptors**

**Two are better than one:** A dual-action ligand, designed to target both integrin α_V_β_3_ and vascular endothelial growth factor receptors (VEGFRs) and possibly block their “crosstalk”, strongly inhibits the VEGF-stimulated morphogenesis in Human Umbilical Vein Endothelial Cells (HUVEC), preventing the formation of new blood vessels.


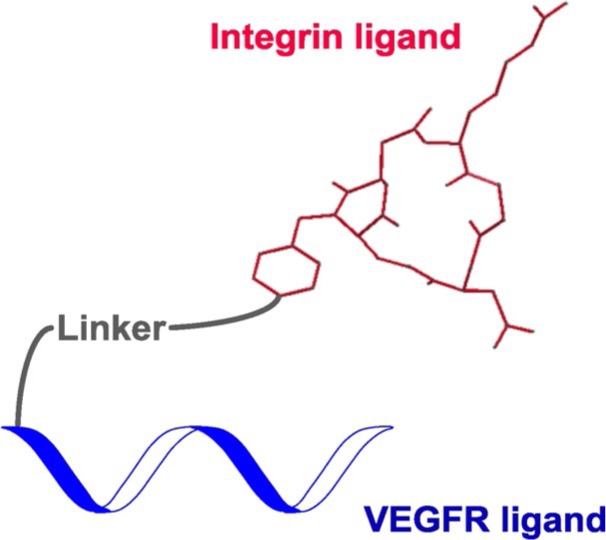


Y. Duan,* C. D. Stinespring, B. Chorpening

642 – 650

**Electronic Structures, Bonding Configurations, and Band-Gap- Opening Properties of Graphene Binding with Low-Concentration Fluorine**

**Buckling and band gaps:** Introducing a band gap in graphene is useful for many applications. Creating defects and covalent binding with other atoms are effective ways to open the band gap. We investigate the structure and impact of low-level fluorine defects on the electrical properties of graphene and show that the band-gap opening depends not only on the fluorine doping level, but also on the configurations of fluorine binding.


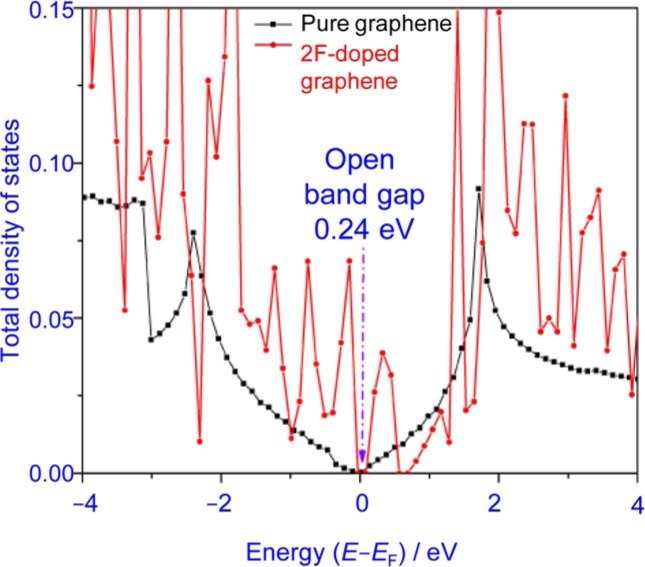


D. MacLeod Carey, T. Gomez, C. Morales-Verdejo, A. MuÇoz-Castro*

651 – 655

**Influence of Ag^+^ on the Magnetic Response of [2.2.2]Paracyclophane: NMR Properties of a Prototypical Organic Host for Cation Binding Based on DFT Calculations**

**Magnetic personality:** Deeper insight into the NMR spectroscopic properties of host–guest systems formed through cation–π interactions is provided by using information from experimentally determined NMR chemical shift values to guide DFT calculations. This approach can be useful for gathering further information regarding local and global variations in NMR shifts for host–guest pairs that involve both inorganic and organic hosts.


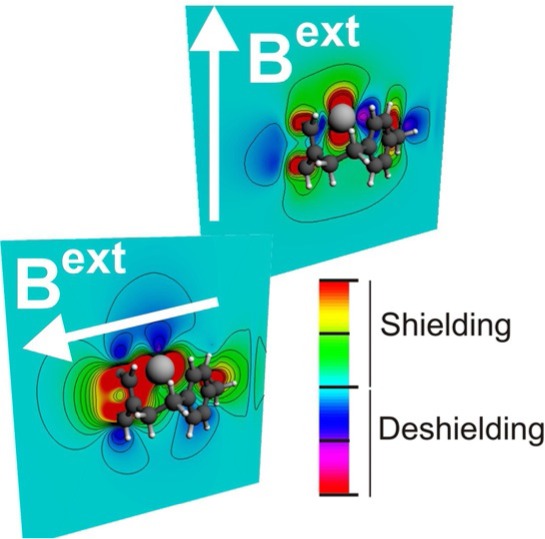


## THESIS TREASURY

M. Ponce-Vargas*

656 – 660

**Metallacycles Capabilities in Host–Guest Chemistry**

**The perfect host for anions!** The nature of host–guest interactions in metallacycle-based complexes was determined by various methodologies. Additionally, by plotting the atomic multipole moment tensors, a clear representation of the higher-order electrostatic interactions within the studied systems, such as dipole–quadrupole and quadrupole– quadrupole forces, was obtained.


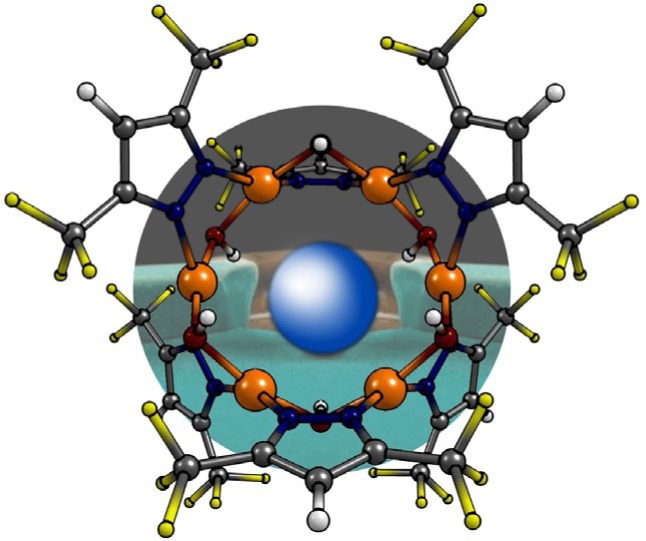


## SERVICE

* Author to whom correspondence should be addressed.

Supporting information is available on the WWW (see article for access details).

A video clip is available as Supporting Information on the WWW (see article for access details).

This is an open-access article, published under the terms and conditions of a Creative Commons License, as stated in the final article.

Contributions labeled with this symbol have been judged as “Very Important Papers” by the referees.

**All the Tables of Contents may be found on the WWW under: http://www.chemistryopen.org**

## CORRIGENDUM

N. Iida, K. Tanaka, E. Tokunaga, H. Takahashi, N. Shibata* Regioisomer-Free C_*4h*_ β-**Tetrakis(tertbutyl) metallo-phthalocyanines: Regioselective Synthesis and Spectral Investigations**

Due to a calculation error, the charge distribution values are incorrect for the CN groups of **3 b**given in Figure 2a; the corrected figure is shown below.


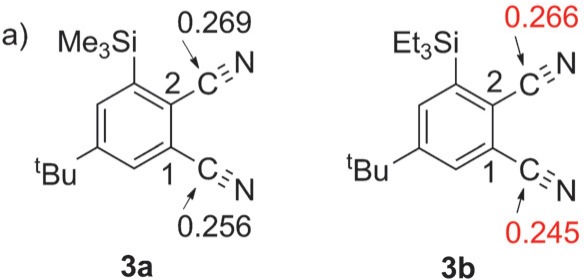


The corresponding discussion on p. 104 should read: “In order to discuss the high C_*4h*_ regioselectivity achieved by our methodology, a computation was attempted next. Hanack reported that the regioselectivity of the formation of phthalocyanines depends on the difference in reactivity between two cyano groups of phthalonitrile.^[8c]^ Hence, the charge distributions of two cyano groups on **3 a** and **3 b** were calculated (DFT/B3LYP/6-31G*) (Figure 2a). In **3 a**, the charge distribution of the cyano group next to the trimethylsilyl group was almost the same as another cyano group (0.269 (C_*2*_) versus 0.256 (C_*1*_)), which indicates that their reactivity is similar. The charge distribution of each cyano group in **3 b** is also almost the same (0.266 (C_*2*_) versus 0.245 (C_*1*_)). These results are consistent with the experimental observation that the regioselectivity by **3 a** and **3 b** are the same. Therefore, the regioselectivity of the observed reaction is presumably caused by the steric effect of the trialkylsilyl group (B >>> B′), while the electronic effect is supplemental (A > A′) (Figure 2b). The steric repulsion between two neighboring trialkylsilyl units on dimer units is the main role for the selectivity. This should be the main reason for the success of the regioselective tetramerization even under very high reaction temperature, while the methodology by Leznoff requires very low reaction temperature due to the reactivity controlled between two cyano groups.^[19]^”

This correction does not influence any of the data presented in the article nor its interpretation. The authors apologize for these mistakes.

